# Psychological experiences of unplanned interruption of patient-controlled intravenous analgesia in adult postoperative patients: A qualitative study

**DOI:** 10.1097/MD.0000000000050578

**Published:** 2026-09-11

**Authors:** Jiao Qin, Wei Zhang, Qiuyue Yang, Ling-yan Gou

**Affiliations:** a Department of Anesthesiology, Affiliated Hospital of North Sichuan Medical College, Nanchong, Sichuan, China.

**Keywords:** patient-controlled intravenous analgesia, postoperative pain, psychological experience, qualitative research, sleep disorders

## Abstract

This qualitative study aimed to explore the psychological experiences of adult postoperative patients who unplanned discontinued patient-controlled intravenous analgesia (PCIA), based on the stress and coping theory and the Activating event, Beliefs, Consequences emotion theory. The findings are intended to provide insights for developing individualized analgesia management and psychological interventions in clinical practice. Employing a purposive sampling strategy, adult patients who experienced unplanned PCIA interruption within 3 days after surgery at a tertiary hospital in Nanchong were recruited. Twenty participants were interviewed. Following 3 pilot interviews, semi-structured interviews were conducted with 16 participants until data saturation was reached; 4 additional interviews were then performed to reinforce the findings. Interview data were analyzed using Giorgi’s descriptive phenomenological method. Two main themes emerged: negative psychological experiences and sleep disturbances. The negative psychological experiences encompassed concerns about increased pain, adverse drug reactions, financial burden, and anxiety regarding the exacerbation of preexisting conditions. Scale-assisted assessments (Visual Analog Scale, Generalized Anxiety Disorder-7, and Insomnia Severity Index) indicated mild to moderate levels of pain, anxiety, and insomnia among some participants. This study is the first to systematically analyze the psychological mechanisms behind unplanned PCIA interruption using a dual-theoretical framework. Negative psychological experiences and sleep disturbances significantly contribute to patients’ decisions to discontinue PCIA prematurely. Clinical staff should prioritize dynamic assessment of patients’ psychological and sleep status. Implementing multidimensional intervention strategies – including optimized physiological analgesia, targeted cognitive education, and behavioral support – is crucial to reduce unplanned PCIA interruption, improve analgesia quality, and enhance postoperative recovery outcomes.

## 1. Introduction

Pain, recognized as the fifth vital sign, is a critical postoperative concern. Studies indicate that approximately 80% of patients experience acute postoperative pain, with 75% reporting moderate to severe intensity.^[1]^ Patient-controlled analgesia is a technique where parameters are set by healthcare professionals, allowing patients to self-administer analgesics on demand.^[2]^ Due to its rapid onset and stable plasma concentration, patient-controlled intravenous analgesia (PCIA) has become a standard method for managing moderate to severe postoperative pain.^[3]^

Nevertheless, unplanned PCIA interruption, which is defined as the discontinuation initiated by the patient or family without prior assessment by an anesthesiologist, is prevalent in clinical practice. A study conducted by Xu et al indicated that 28.2% of patients prematurely terminated PCIA due to apprehensions regarding addiction or side effects, whereas 29.1% discontinued it on account of adverse reactions such as dizziness, nausea, or vomiting.^[4]^ Such unplanned interruptions can undermine analgesia, intensify pain, induce anxiety and insomnia, extend the duration of hospitalization, and augment healthcare costs.

Previous research has predominantly focused on physiological factors influencing PCIA interruption, such as drug dosage, device failure, and surgical type,^[5,6]^ with limited exploration of patients’ psychological cognitions and decision-making processes. The importance of integrating mental health into overall health management is widely recognized in global health strategies.^[7]^ Evidence suggests that systematic psychological interventions can improve patient treatment responses and outcomes.^[8]^

The Activating event, Beliefs, Consequences (ABC) emotion theory posits that an individual’s emotional and behavioral reactions are determined by their cognitions and beliefs about an event.^[9]^ The stress and coping theory highlights the identification and management of stressors.^[10]^ Integrating these frameworks, postoperative pain or drug side effects act as stressors; after cognitive appraisal, they may provoke emotions such as anxiety and worry, ultimately leading to pump discontinuation. Therefore, this qualitative study aims to explore the psychological experiences of adult patients who unplanned discontinued PCIA, providing a foundation for developing integrated biopsychosocial pain management protocols.

## 2. Materials and methods

### 2.1. Research materials

A purposive sampling method was employed. Participants were adult patients who underwent surgery, received PCIA, and experienced unplanned analgesia interruption within 3 days postoperatively at a tertiary hospital in Nanchong between October 2024 and January 2025. Inclusion criteria were age ≥18 years; postoperative PCIA use; clear consciousness, normal cognition, and unimpaired communication; complete clinical data; and provision of informed consent. Exclusion criteria were requesting discontinuation before any PCIA use; age <18 years; presence of psychiatric illness, cognitive impairment, or communication difficulties. The study was approved by 2024 Nanchong Social Science Planning Project (NC24B273) and 2025 Nanchong Social Science Planning Project (NC25B226).

All participants provided written informed consent. Sampling continued until informational saturation was achieved. Twenty participants were ultimately included, with the first 3 serving as a pilot. Formal interviews with 16 participants achieved saturation, and 4 additional interviews were conducted to strengthen the robustness of the findings.

General information of the respondents is shown in Table 1.

**Table 1:** General characteristics of participants (n = 20).

ID	Gender	Age (yr)	Department	Reasons for interruption	Feelings after interruption	VAS score	GAD-7 score	ISI score
Z1	Female	72	Orthopedics	Vomiting	Vomiting relieved	3	1	8
Z2	Male	67	Thoracic surgery	Dizziness	Dizziness relieved	2	0	8
Z3	Female	69	Thoracic surgery	Dizziness, vomiting	Dizziness, vomiting relieved	2	2	0
Z4	Female	56	Thoracic surgery	Dizziness	Dizziness relieved	1	0	6
Z5	Male	45	Hepatobiliary surgery	Drowsiness	Mentally clear	1	19	6
Z6	Female	56	Orthopedics	Severe vomiting	Wound pain	4	8	7
Z7	Female	55	Thyroid and breast surgery	Severe vomiting	Wound pain	4	3	12
Z8	Male	58	Orthopedics	Severe vomiting	Vomiting relieved	2	0	6
Z9	Female	42	Obstetrics	No pain perceived	No pain or discomfort	1	4	0
Z10	Male	58	Thoracic surgery	Drowsiness, dizziness	Gradually mentally clear, dizziness relieved	3	0	9
Z11	Female	67	Orthopedics	Dizziness	Dizziness relieved	2	6	9
Z12	Female	62	Thoracic surgery	Dizziness	Dizziness relieved	4	0	11
Z13	Female	31	Otorhinolaryngology	Dizziness, nausea	Dizziness relieved	2	1	5
Z14	Female	36	Thoracic surgery	No pain perceived	No pain or discomfort	1	1	3
Z15	Female	48	Thoracic surgery	Vomiting	Vomiting relieved	2	5	7
Z16	Female	59	Gynecology	Vomiting	Vomiting relieved	2	7	11
Z17	Female	67	Thoracic surgery	Triggered preexisting condition, dizziness, vomiting	Dizziness still severe, vomiting relieved	3	11	12
Z18	Female	63	Thoracic surgery	Dizziness, vomiting	Dizziness, vomiting still severe	2	0	6
Z19	Female	67	Thoracic surgery	Psychological discomfort	Wound pain	4	3	3
Z20	Female	73	Maxillofacial surgery	Family worried about side effects	Wound pain	3	5	8

VAS: 0 = no pain; 1 to 3 = mild pain; 4 to 6 = moderate pain; ≥7 = severe pain.

GAD-7: ≤4 = no anxiety; 5 to 9 = mild anxiety; 10 to 14 = moderate anxiety; ≥15 = severe anxiety.

ISI: ≤7 = normal; 8 to 14 = mild insomnia; 15 to 21 = moderate insomnia; ≥22 = severe insomnia.

Vomiting and dizziness were the primary reasons for interruption (each 45% among reasons reported). The median scores (IQR) were VAS, 2.0 (1.0–3.0); GAD-7, 1.5 (0–6.5); ISI, 7.0 (5.0–9.0).

GAD-7 = Generalized Anxiety Disorder-7, ID = identification, ISI = Insomnia Severity Index, IQR = interquartile range, VAS = Visual Analog Scale.

### 2.2. Methods

#### 2.2.1. Interview guide design

Based on a literature review and research team discussions, a semi-structured interview guide was developed. The guide was revised and refined following 3 pilot interviews, where issues such as ambiguous phrasing were identified. The final guide covered the following dimensions: primary concerns before surgery; pain experience during PCIA use; main reasons for PCIA interruption; current discomforts.

#### 2.2.2. Research tools

To aid in interpreting subjective descriptions, participants were guided to complete the Visual Analog Scale for pain intensity, the Generalized Anxiety Disorder-7 scale for anxiety symptoms, and the Insomnia Severity Index for sleep disturbances during the interview. These served as auxiliary assessment tools, not as primary outcome measures. All scales have been validated in domestic research with good reliability and validity (Cronbach’s alpha > 0.8).

#### 2.2.3. Data collection

Prior to the interview, the researcher explained the study’s purpose, methods, and significance to participants and obtained written consent. Interviews were conducted within 24 hours after PCIA interruption. Semi-structured, face-to-face interviews were carried out one-on-one in a quiet, private room, lasting 15 to 20 minutes, and were audio-recorded. Interviews were anonymized using codes. After each interview, a summary was reviewed with the participant to ensure accuracy. Data collection ceased when thematic saturation was reached (after 16 interviews), with 4 subsequent interviews confirming no new themes emerged.

### 2.3. Data analysis

Interview recordings were transcribed verbatim within 24 hours. Textual data were managed using NVivo 12.0 (QSR International Pty Ltd.). Analysis followed Giorgi’s 4-step phenomenological method: reading transcripts for an overall sense; identifying meaning units related to psychological experience; transforming meaning units into themes; synthesizing themes to construct a structured description of the psychological experience. Coding was performed independently by 2 researchers, with discrepancies resolved through team discussion.

## 3. Results

Analysis of interview data from 20 adult patients with unplanned PCIA interruption yielded 2 main themes and associated sub-themes.

### 3.1. Negative psychological experiences

#### 3.1.1. Distress from increased postoperative pain

Patients with larger wounds or higher pain sensitivity reported intensified pain after stopping the pump, as side effects diminished. Visual Analog Scale scores ranged from 1 to 4 (mild to moderate). Representative quotes are as follows. Z6: “Right after surgery, I stopped the pump, and the pain was so bad I didn’t know how to position my leg.” Z7: “My left-side wound hurt after surgery. I could only sit, not lie down, and had to move slowly.” Z19: “It hurt even to breathe. Sitting was better, but I still couldn’t move much.”

#### 3.1.2. Anxiety over adverse drug reactions

Dizziness, nausea, vomiting, and drowsiness were common reasons for interruption. Z16: “What could I do? I kept vomiting. It got a bit better after stopping the pump, but I still felt terrible.” Concerns from family members also influenced decisions, consistent with findings from Kim et al.^[6]^ Z20: “My family wanted me to use the pump, but I read online about side effects, so I turned it off now.” Z5 described significant drowsiness: “After using this medicine, I would fall back asleep immediately if woken up. The doctor stopped the pump, and I still feel groggy.”

#### 3.1.3. Anxiety about exacerbating preexisting conditions

A few patients worried that PCIA might trigger underlying health issues, increasing psychological burden. Z17: “After using this, she vomited and felt dizzy, which brought back her old vertigo. She couldn’t lie down, only sit, and felt very dizzy.”

#### 3.1.4. Concerns about financial burden

Financial considerations were a significant psychological factor, aligning with findings from Chou et al^[11]^ and interacting with anxieties about underlying conditions. Z9: “How much does this medicine cost? I haven’t used it; can I get a refund?” Z14: “I’m not in pain now. Can I get a refund for the leftover medicine? Medical expenses are high, and the financial pressure is big.”

### 3.2. Sleep disturbances under multifactor interaction

Forty-five percent of patients experienced mild to severe sleep disturbances (Insomnia Severity Index scores 8–28). The correlation between drug side effects, anxiety, and sleep problems was notable. Z7: “Just finished vomiting, then chest pain started, plus dizziness—I couldn’t sleep well at all.” Z12: “I felt very dizzy. I stopped the medicine. I was in pain and worried about my condition, so I couldn’t fall asleep until early morning.”

## 4. Discussion

This study reveals that unplanned PCIA interruption is closely associated with patients’ negative psychological experiences and sleep disturbances, highlighting the interaction of biopsychosocial factors. Based on the stress and coping theory and ABC emotion theory, we propose the following multilevel intervention strategies.

**Table:** 

Intervention level	Comprehensive intervention strategy recommendations based on findings
1. Physiological	Address increased postoperative pain and adverse drug reaction risks by blocking pain stressors and strengthening individualized assessment and dynamic protocol adjustment. Strategies include prophylactic antiemetics^[12]^; low-dose infusion modes^[13]^; adjuvant non-pharmacological therapies (e.g., auriculotherapy,^[14]^ facial acupuncture^[15]^); and the application of multimodal analgesia.^[16]^
2. Cognitive	Implement in 2 stages: preliminary education 1 day preoperatively via video (15 min) + one-on-one Q&A (5 min), reinforced within 24 h postoperatively. Deliver personalized education to dispel misconceptions: systematically explain PCIA mechanics; present evidence-based data on the real incidence and controllability of side effects; clarify the extremely low addiction risk with short-term postoperative use; explain the necessity of cost components. Use multiple channels (videos, social media, face-to-face) to educate patients and families on surgical procedures, drug effects, and side effect management. Continuously assess understanding and unresolved concerns to ensure effective cognitive realignment.
3. Psychological and behavioral	Relaxation training (low-cost, easy to implement): deep breathing, aerobic exercise, meditation.^[17]^ Non-pharmacological sleep improvement: cognitive behavioral therapy for insomnia,^[18]^ music therapy,^[19]^ aromatherapy.^[20]^ (3) Application of new technologies: utilize virtual reality to simulate recovery scenarios, alleviating psychological stress and improving sleep.^[21]^

## 5. Conclusion

Postoperative patients’ concerns regarding pain, drug side effects, financial burden, and worsening of preexisting conditions, coupled with associated sleep disturbances, significantly increase the risk of unplanned PCIA interruption. The stress and coping theory explains the negative impact of pain as a stressor on emotions and sleep, while the ABC emotion theory suggests that modifying irrational cognitions can improve emotional and behavioral responses. Clinical practice should adopt integrated, individualized analgesia management encompassing physiological, cognitive, and psychological-behavioral dimensions to reduce the rate of unplanned interruption and enhance analgesia safety and patient satisfaction.

This study is a single-center qualitative study with a relatively small sample size, limiting the generalizability of the findings. As patient long-term recovery outcomes were not followed up, subsequent multicenter cohort studies are needed for validation.

## Author contributions

**Conceptualization:** Jiao Qin.

**Formal analysis:** Wei Zhang.

**Investigation:** Wei Zhang.

**Software:** Qiuyue Yang.

**Writing – original draft:** Jiao Qin, Qiuyue Yang.

**Writing – review & editing:** Ling-yan Gou.
